# Epigenetic features improve TALE target prediction

**DOI:** 10.1186/s12864-021-08210-z

**Published:** 2021-12-29

**Authors:** Annett Erkes, Stefanie Mücke, Maik Reschke, Jens Boch, Jan Grau

**Affiliations:** 1 Institute of Computer Science, Martin Luther University Halle-Wittenberg, Halle, Germany; 2 Institute of Plant Genetics, Leibniz Universität Hannover, Hannover, Germany

**Keywords:** Transcription activator-like effector, Computational prediction, Chromatin, DNA methylation

## Abstract

**Background:**

The yield of many crop plants can be substantially reduced by plant-pathogenic *Xanthomonas* bacteria. The infection strategy of many *Xanthomonas* strains is based on transcription activator-like effectors (TALEs), which are secreted into the host cells and act as transcriptional activators of plant genes that are beneficial for the bacteria.The modular DNA binding domain of TALEs contains tandem repeats, each comprising two hyper-variable amino acids. These repeat-variable diresidues (RVDs) bind to their target box and determine the specificity of a TALE.All available tools for the prediction of TALE targets within the host plant suffer from many false positives. In this paper we propose a strategy to improve prediction accuracy by considering the epigenetic state of the host plant genome in the region of the target box.

**Results:**

To this end, we extend our previously published tool PrediTALE by considering two epigenetic features: (i) chromatin accessibility of potentially bound regions and (ii) DNA methylation of cytosines within target boxes. Here, we determine the epigenetic features from publicly available DNase-seq, ATAC-seq, and WGBS data in rice.We benchmark the utility of both epigenetic features separately and in combination, deriving ground-truth from RNA-seq data of infections studies in rice. We find an improvement for each individual epigenetic feature, but especially the combination of both.Having established an advantage in TALE target predicting considering epigenetic features, we use these data for promoterome and genome-wide scans by our new tool EpiTALE, leading to several novel putative virulence targets.

**Conclusions:**

Our results suggest that it would be worthwhile to collect condition-specific chromatin accessibility data and methylation information when studying putative virulence targets of *Xanthomonas* TALEs.

**Supplementary Information:**

The online version contains supplementary material available at (10.1186/s12864-021-08210-z).

## Background

The cultivation of crop plants can be severely impaired by the infestation with phytopathogenic *Xanthomonas* bacteria. In many parts of the world, the crop yield of rice plays a key role in ensuring nutrition of the population. However, the yield of a rice field can be substantially reduced due to infection with *Xanthomonas oryzae* pv. *oryzae* (*Xoo*) or *Xanthomonas oryzae* pv. *oryzicola* (*Xoc*), which cause significant loss in many cultivation areas [1].

Host plant infection depends on the bacterial type III secretion system. Specific bacterial effector proteins are secreted into the plant cell, where they modulate plant response. Of these, transcription activator-like effectors (TALEs) are sequence-specific DNA-binding proteins that bind to host promoters to activate the expression of downstream genes. If such genes promote disease, they are termed susceptibility genes.

TALE proteins comprise a nuclear localization signal, a modular DNA-binding domain, and an activation domain. The DNA-binding domain of natural TALEs is composed of 1.5 to 33.5 consecutive repeats, where each repeat binds to one nucleotide of the target box [2, 3]. Each repeat com- prises ∼ 34, highly conserved, amino acids (AAs). Only the residues at position 12 and 13 are hyper-variable and are called repeat-variable diresidue (RVD). The second residue of the RVD binds to the target base, while the first residue has a stabilizing effect [4, 5].

The target boxes of a TALE can be predicted based on the one-to-one correspondence between RVD and target base [3, 6]. For example, the RVD HD (His and Asp) prefers to bind to base ’C’. Furthermore, TALE target boxes show an additional preference for the base at “position 0” directly preceding the nucleotides bound by the repeat array, which is usually ‘T’ [3, 7]. As a rare exception, individual aberrant repeats of unusual length may loop out of the repeat array to allow binding to a target DNA sequence that is one bp shorter [8].

As shown recently [9, 10], DNA methylation alters the preference of RVDs for cytosines. DNA methylation is an epigenetic mechanism, where a methyl group is added to cytosine to form 5-methylcytosine (5mC) [11]. Biochemical analyses [9] showed that methylated C is bound by NG rather than HD. The RVD NG binds specifically to base T, which is structurally identical to 5mC in the part that faces the major grove of the DNA which essentially is bound by the RVD [12]. Hence, the RVD NG may also bind well to 5mC in addition to T. The RVD N*, where ‘*’ represents the deletion of the 13th amino acid, is known to preferentially bind to T or C, and has been shown to also bind to 5mC and 5hmC [13].

Furthermore, it has been shown that the accessibility of chromatin in the region of the target box has an impact on the binding ability of TALEs [12, 14].

Several tools to identify potential target boxes based on the RVD sequence exists. These include the “Target Finder” of TALE-NT suite (http://tale-nt.cac.cornell.edu/) [15, 16], the tool Talvez (http://bioinfo-web.mpl.ird.fr/cgi-bin2/talvez/talvez.cgi) [17] and TALgetter (http://www.jstacs.de/index.php/TALgetter) [18].

Our recently published tool PrediTALE (http://www.jstacs.de/index.php/PrediTALE) [19] models binding specificities based on quantitative data and includes further aspects of the binding of TALEs to their target boxes. It considers putative dependencies between adjacent RVDs and dependencies between the first RVD and the preference at position 0 of the target box, as well as the frame shift that may occur for aberrant repeats [8]. Prediction of TALE targets with PrediTALE achieves an improved prediction accuracy compared with previous approaches.

Still, the predictions of all tools suffer from many false positives. All existing tools neglect epigenetic features when predicting TALE target boxes, although DNA methylation and chromatin accessibility are known to be important determinants of TALE-DNA binding. Hence, we aim at improving the accuracy of TALE target prediction by incorporating such epigenetic features into an extended version of PrediTALE termed EpiTALE.

Our new application suite EpiTALE contains all tools necessary for TALE target prediction incorporating epigenetic features of the target site. First, we extend the PrediTALE model to consider DNA methylation information when making predictions and, second, we filter predictions using accessibility data such as DNase-seq and ATAC-seq.

We approximate the specificities of the different RVD types for methylated cytosine based on experimental data. Users of our new suite EpiTALE may then provide methylation data in addition to genomic sequence or extracted promoters, which will be considered in prediction scoring. EpiTALE is the first approach that accounts for methylated cytosine when predicting TALE target boxes.

To incorporate chromatin accessibility, we annotate the chromatin accessibility of target sites predicted by EpiTALE using DNase-seq or ATAC-seq data and suggest criteria to filter putatively inaccessible target boxes.

We benchmark EpiTALE based on RNA-seq data after *Xanthomonas* infection of rice plants. Here, we consider infection studies for 3 *Xoo* and 10 *Xoc* strains, where each strain expresses a different repertoire of TALEs, with up to 27 TALEs per strain [20–23].

We further apply EpiTALE using both, methylation information and a filter based on chromatin accessibility, for genome-wide predictions and identify previously neglected putative TALE target boxes, which show a transcription response in infection experiments according to RNA-seq data.

## Methods

### Data

#### Bisulfite sequencing data of rice

We obtained publicly available whole genome bisulfite sequencing (WGBS) data of rice from the European Nucleotide Archive (ENA) https://www.ebi.ac.uk/ena available under run accessions SRR3485276 (replicate 1) and SRR3485277 (replicate 2). These data have been collected as part of a study by Zheng et al. [24], who investigated epigenetic changes under drought stress. The two WGBS runs we consider in this study to determine DNA methylation levels in rice correspond to two biological replicates of Huhan3 (O. *sativa* L. ssp. *japonica*) under normal conditions.

We clipped sequencing adapters and removed low-quality bases at the ends of these paired end reads using Trimmomatic (v0.33) [25] with parameters “CROP:80 SLIDINGWINDOW:4:28 MINLEN:20”. We mapped the processed reads to the rice genome (MSU7, http://rice.uga.edu/pub/data/Eukaryotic_Projects/o_sativa/annotation_dbs/pseudomolecules/version_7.0/all.dir/all.chrs.con) via Bismark (v0.20.0) [26] and Bowtie2 (v2.3.4.3) [27]. We used the deduplication tool from Bismark to remove PCR artefacts.

We used the Bismark methylation extractor to determine the methylation levels and set the following parameters “–bedGraph –CX -p”, which reports methylation of cytosines in the three contexts ‘CpG’, ‘CpHpG’ and ‘CpHpH’ (H = ‘A’, ‘C’ or ‘T’). The output contains a coverage file reporting count values for methylated and unmethylated cytosines. We finally merged the coverage files of both replicates by simply summing the (unnormalized) counts at identical positions and then computing the methylation level. To obtain conservative methylation calls, we introduced a bias towards unmethylated cytosines in sparsely covered regions by adding a pseudo count of 1 to the count values for unmethylated cytosines.

Supplementary Figure S1 has been generated by the methylation report of ViewBS [28], and shows the distribution of methylation levels and the global methylation level of the three methylation contexts.

#### RNA-seq data

To benchmark EpiTALE, we used RNA-seq data as described previously [19]. Briefly, we used in-house RNA-seq infection studies of rice leaves with *Xoo* strains PXO83, PXO142, ICMP 3125^T^ and publicly available data from infection studies with *Xoc* strains BLS256, BLS279, CFBP2286, B8-12, L8, RS105, BXOR1, CFBP7331, CFBP7341, CFBP7342 [22]. Genes that are differentially expressed in the infection studies compared to mock control and whose promoters contain a putative target box of a TALE are defined as true positive targets. A direct assignment to a single TALE of a strain is not possible based on the RNA-seq data, since the entire TALE repertoire of a strain acts simultaneously in the infection studies.

#### DNase-seq and ATAC-seq data

To identify accessible regions, we mapped publicly available DNase-seq and ATAC-seq data to the rice genome (MSU7). Neither of these libraries has been collected under conditions perfectly matching the RNA-seq data from the infection studies. However, chromatin accessibility might still be sufficiently similar to be informative for subsequent TALE target prediction.

We downloaded DNase-seq reads of rice seedlings [29] from NCBI Sequence Read Archive (SRA), accession SRX038423, and used Cutadapt [30] for clipping sequencing adapters and Trimmomatic (v0.33) [25] with parameters “SLIDINGWINDOW:4:20 MINLEN:20” for removing low-quality bases. We mapped the reads to the rice MSU7 genome using Bowtie2 [27]. In the following we will refer to this DNase-seq dataset as ’DNase’ to improve readability. Two ATAC-seq datasets for wildtype rice are publicly available. In the first study, nucleosome- free chromatin of 14 day old rice leaves was measured in a time series under different stress conditions [31]. As we are interested in normal conditions, we only consider the control experiments from the corresponding ENA archive (accession: PRJNA305365). We refer to this dataset as ’ATAC1’.

For the second study [32], ATAC-seq data of rice nuclei from leaf mesophyll cells where downloaded from ENA (accession: PRJNA391551) and we refer to this dataset as ’ATAC2’.

For both datasets we used Trimmomatic (v0.39) in paired end mode for clipping sequencing adapters and for removing low-quality bases from the ends of reads with parameters “ILLUMINACLIP:NexteraPE-PE.fa:2:30:10 SLIDINGWINDOW:4:20 MINLEN:20”, mapped the resulting reads with Bowtie2 to rice genome (MSU7) and removed duplicates with Samtools [33].

The mapping statistics of all three datasets are summarized in Supplementary Table A. The two ATAC-seq datasets have rather low numbers of uniquely mapped reads. Especially the ATAC1 dataset has only ∼ 5*%* uniquely mapped reads. Hence, we decided to use only DNase and ATAC2 for benchmarking.

For both, the DNase and the ATAC2 dataset we used JAMM [34] for peak calling with parameters “-f 1 -d y”, which results in JAMM only considering 5’ ends of reads and retaining duplicate reads.

We used the open-source library Jstacs [35, 36] (class projects.encodedream.Pileup) to calculate the 5’ coverage with ATAC-seq or DNase-seq reads at each position and normalized coverage relative to the mean of a 10000 bp sliding window.

### Model

The statistical model behind EpiTALE is based on modelling the total binding score of a putative target box ***x***=*x*_0_*x*_1_…*x*_*L*_ to the RVD sequence ***r***=*r*_1_*r*_2_…*r*_*L*_ of length *L*. Each RVD *r*_*ℓ*_∈{*AA*,…,*YY,A*∗,…,*Y*∗} is composed of its two amino acids. Each putative target box is a sequence of *x*_*ℓ*_∈{*A,C,G,T*}. Since it is known that the 13th AA of an RVD has the largest contribution to nucleotide preference [4, 5], and since quantitative data about binding preference are sparse for many RVDs, the EpiTALE model considers all RVDs with the same 13th AA as identical by default. For a subset of RVDs with sufficient data, however, the model explicitly considers the 12th AA of an RVD as well.

In addition to the original definition of the PrediTALE model, we introduce *q*_*ℓ*_∈[0,1] as the methylation probability at position *ℓ* and ***q***∈*q*_1_…*q*_*L*_ as the sequence of methylation probabilities for each nucleotide of the target box ***x***. These methylation probabilities are considered by the EpiTALE model for all nucleotides that are directly bound by an RVD of a TALE.

In the following, we provide the definition of the complete EpiTALE model and highlight the differences to the original PrediTALE model.

The total binding score of a putative target box ***x*** given the RVD sequence ***r*** of a TALE and the methylation probabilities ***q*** is modelled as the sum of the following terms: i) the score *m*_0_(*x*_0_|*r*_1_,***θ***_0_) of the zero-th nucleotide *x*_0_ given the first RVD *r*_1_, ii) the score $m_{1}\left (x_{1} | r_{1}, \boldsymbol {\theta }_{1}, \boldsymbol {\theta }_{m}, \boldsymbol {\theta }^{M}_{m}, q_{1}\right)$ for binding between first RVD *r*_1_ and first nucleotide *x*_1_, and iii) the score $m\left (x_{\ell } | r_{\ell -1}, r_{\ell }, \boldsymbol {\theta }_{m}, \boldsymbol {\theta }^{M}_{m},q_{\ell }\right)$ for binding of the remaining RVDs to the remaining nucleotides. The terms ii) and iii) may be weighted by a position-dependent but sequence-independent term *p*(*ℓ*|***θ***_*p*_). In the EpiTALE model, the terms ii) and iii) further depend on the methylation probability *q*_*ℓ*_ of nucleotide *x*_*ℓ*_:

$${}\begin{aligned} s(\boldsymbol{x} | \boldsymbol{r}, \boldsymbol{q}, \boldsymbol{\theta}) &= m_{0}(x_{0} | r_{1}, \boldsymbol{\theta}_{0}) \\ & \quad+m_{1}(x_{1} | r_{1}, \boldsymbol{\theta}_{1}, \boldsymbol{\theta}_{m}, \boldsymbol{\theta}^{M}_{m}, q_{1}) \cdot p(1 | \boldsymbol{\theta}_{p}) \\ & \quad+\sum_{\ell=2}^{L} m(x_{\ell} | r_{\ell-1}, r_{\ell}, \boldsymbol{\theta}_{m}, \boldsymbol{\theta}^{M}_{m},q_{\ell}) \cdot p(\ell | \boldsymbol{\theta}_{p}) \end{aligned} $$

The set of real-valued parameters $\boldsymbol {\theta } = \left (\boldsymbol {\theta }_{0}, \boldsymbol {\theta }_{1}, \boldsymbol {\theta }_{m},\boldsymbol {\theta }^{M}_{m}, \boldsymbol {\theta }_{p}\right)$ includes the parameters for binding to the zero-th, first and remaining nucleotide, the binding specifities for 5mC as well as the position-dependent term.

In the following, we provide a detailed definition of each of the individual terms of the EpiTALE model.

As in the original PrediTALE model, the term for binding to the zero-th nucleotide *m*_0_(*x*_0_|*r*_1_,***θ***_0_) is independent of methylation probabilities, since there are no appropriate activity or binding studies regarding methylation sensitivity available, yet.

As in PrediTALE, this term corresponds to the sum of the following parameter values: i) the *a-priori* parameter of nucleotide zero $\pi _{x_{0}}$, ii) the parameter $\theta _{0,x_{0}}$ for the zero-th nucleotide. In addition, we define a set $\mathcal {R}_{0}$ of all RVDs with sufficient data to model the dependency of the nucleotide preference at position zero on the first RVD. In case that the first RVD *r*_1_ is in this set $\mathcal {R}_{0}$, we further add the parameter $\theta _{0,x_{0}|r_{1}}$ for the zero-th nucleotide depending on *r*_1_:

$$\begin{array}{@{}rcl@{}} m_{0}(x_{0} |r_{1}, \boldsymbol{\theta}_{0}) &=& \pi_{x_{0}} + \theta_{0,x_{0}} + \delta(r_{1} \in \mathcal{R}_{0})\cdot \theta_{0,x_{0}|r_{1}}. \end{array} $$

Here, we define $\mathcal {R}_{0} = \{ HD,NN,NG,NI,NS \}$ and *π*_*T*_= log(0.6),*π*_*C*_= log(0.3),*π*_*A*_=*π*_*G*_= log(0.05).

The binding of the first RVD to the first nucleotide of the target box is modelled by the term $m_{1}(x_{1} | r_{1}, \boldsymbol {\theta }_{1}, \boldsymbol {\theta }_{m}, \boldsymbol {\theta }^{M}_{m}, q_{1})$, which consists of the sum of two terms, one for the unmethylated state and one for the methylated state of nucleotide *x*_1_.

Specifically, the probability (1−*q*_1_) that the first position is unmethylated is multiplied by the term adopted from the original PrediTALE model, which always contains a parameter $\theta _{m,x_{1} | r_{1,13}}$ for the general preference of the 13th AA *r*_1,13_ of the RVD to nucleotide *x*_1_. For a subset $\mathcal {R}_{1}$ of RVDs, a parameter $\theta _{m,x_{1} | r_{1}}$ is added that models the dependency on the complete RVD. For a second subset $\mathcal {R}_{2}$ of RVDs, a parameter $\theta _{1,x_{1} | r_{1,13}}$ captures a deviating nucleotide preference of the 13th AA of the RVD that is specific to position 1 of the target box. The sets $\mathcal {R}_{1}$ and $\mathcal {R}_{2}$ are again chosen according to the availability of sufficient data.

Given a methylation probability *q*_1_>0 at position 1, this methylation probability is multiplied by the parameter $\theta ^{M}_{m,x_{1} | r_{1,13}}$ modelling the preference of the 13th AA of the first RVD to bind to a methylated cytosine. If the first RVD *r*_1_ belongs to the set $\mathcal {R}_{1}$, the general preference $\theta ^{M}_{m,x_{1} | r_{1}}$ of the complete first RVD to bind methylated cytosine is added. In the methylated case, data are not sufficient to also determine parameters specific to position 1, and the third parameter in analogy to the unmethylated case is omitted.

Combining all these terms into the formal definition of $m_{1}(x_{1} | r_{1}, \boldsymbol {\theta }_{1}, \boldsymbol {\theta }_{m}, \boldsymbol {\theta }^{M}_{m}, q_{1})$, we obtain

$${}\begin{aligned} & m_{1}\left(x_{1} | r_{1}, \boldsymbol{\theta}_{1}, \boldsymbol{\theta}_{m}, \boldsymbol{\theta}^{M}_{m}, q_{1}\right)\\ &\quad= (1-q_{1})\cdot \left[\theta_{m,x_{1} | r_{1,13}} + \delta(r_{1} \in \mathcal{R}_{1})\cdot\theta_{m,x_{1} | r_{1}}\right.\\ & \quad\quad\left.+ \delta(r_{1,13} \in \mathcal{R}_{2})\cdot \theta_{1,x_{1} | r_{1,13}}\right] \\ & \quad\quad+ q_{1} \cdot \left[\theta^{M}_{m,x_{1} | r_{1,13}}+ \delta(r_{1} \in \mathcal{R}_{1})\cdot\theta^{M}_{m,x_{1} | r_{1}}\right]. \end{aligned} $$

The subsets of RVDs are set to $\mathcal {R}_{1} = \{ HD,NN,NG,HG,NI,NK \}$ and $\mathcal {R}_{2} = \{ D, N, G, I \}$ as originally proposed for PrediTALE [19], and are also used in the remaining, third term of the model that is described in the following.

The binding to the remaining positions is modelled by terms $m\left (x_{\ell } | r_{\ell -1}, r_{\ell }, \boldsymbol {\theta }_{m}, \boldsymbol {\theta }^{M}_{m},q_{\ell }\right)$, which are identical to the previous PrediTALE variant in the unmethylated case. Again, we have a term $\theta _{m,x_{\ell } | r_{\ell,13}}$ for the binding preference of the 13th AA of the RVD. For RVDs with sufficient data (i.e. those in $\mathcal {R}_{1}$), a term $\theta _{m,x_{\ell } | r_{\ell }}$ for the preference of the complete RVD is added. For RVDs with sufficient data to also model dependencies between adjacent RVDs (i.e., those in $\mathcal {R}_{3}$), we include a third term $\theta _{m,x_{\ell } | r_{\ell }, r_{\ell -1,12}}$, where the nucleotide preference depends on the current RVD and the 12th AA of the previous RVD as in the original PrediTALE model.

In case of a methylation probability *q*_*ℓ*_>0, the preference $\theta ^{M}_{m,x_{\ell } | r_{\ell,13}}$ of the 13th amino acid to bind a methylated cytosine and, if applicable, the preference $\theta ^{M}_{m,x_{\ell } | r_{\ell }}$ of the entire RVD for a methylated cytosine are included. Again, data for the methylated case are not sufficient to also include a term modelling dependencies between adjacent RVDs, and this term in analogy to the unmethylated case is omitted. Hence, we obtain the complete definition of $m\left (x_{\ell } | r_{\ell -1}, r_{\ell }, \boldsymbol {\theta }_{m}, \boldsymbol {\theta }^{M}_{m},q_{\ell }\right)$ as

$${}\begin{aligned} &m\left(x_{\ell} | r_{\ell-1}, r_{\ell}, \boldsymbol{\theta}_{m}, \boldsymbol{\theta}^{M}_{m},q_{\ell}\right) \\ &\quad= (1-q_{\ell})\cdot \left[\theta_{m,x_{\ell} | r_{\ell,13}} + \delta(r_{\ell} \in \mathcal{R}_{1})\cdot \theta_{m,x_{\ell} | r_{\ell}}\right.\\ &\quad\quad\left.+ \delta(r_{\ell},r_{\ell-1} \in \mathcal{R}_{3})\cdot \theta_{m,x_{\ell} | r_{\ell}, r_{\ell-1,12}}\right]\\ &\quad\quad+ q_{1} \cdot \left[\theta^{M}_{m,x_{\ell} | r_{\ell,13}} + \delta(r_{\ell} \in \mathcal{R}_{1})\cdot \theta^{M}_{m,x_{\ell} | r_{\ell}}\right]. \end{aligned} $$

In analogy to the original PrediTALE publication, we set $\mathcal {R}_{3} = \{ HD,NN,NG,NI \}$.

We set *q*_*ℓ*_:=0, if the nucleotide at position *ℓ* of the target sequence is not a cytosine.

### Scale parameters to model specificities for 5mC

As described in the previous section, we extended the previously trained PrediTALE model by adding parameters for the specificity to bind to ’5mC’ to incorporate methylation information into the TALE target prediction of EpiTALE. The former training [19] included pairs of TALEs and their putative target boxes from different experiments [37–41].

A thorough study by Zhang *et al.* tested all theoretically possible combinations of RVDs to bind to 5-methylcytosine (5mC), 5-hydroxymethylcytosine (5hmC), cytosine and thymine [10]. For this purpose, the activation of a GFP expression reporter was measured in the screening.

To obtain fitted values for the parameters $\theta ^{M}_{m}$ (see above) representing binding preferences to methylated cytosines, we considered specificities for methylated cytosines determined by Zhang *et al.* [10]. To this end, we scaled the measured values from Zhang *et al.* to match the range of parameter values of the original PrediTALE model. Specifically, we used the two reference points for cytosine and thymine also present in the data of Zhang *et al.* to scale the raw measured values $\omega _{x_{\ell } | r_{\ell }}$ to fit to our trained parameter space.

Let $\omega _{x_{\ell } | r_{\ell,13}}$ be the arithmetic mean of all $\omega _{x_{\ell } | r_{\ell }}$ with the same 13th AA.

The specificity of the 13th AA of the RVD to bind 5mC is determined by the following scaling:

$$\theta^{M}_{m,5mC | r_{\ell,13}}=\omega_{5mC | r_{\ell,13}}\cdot a + b $$

with

$$\begin{aligned} a &= \frac{\max_{s \in \{C,T\}} \theta_{m,s | r_{\ell,13}} -\min_{s \in \{C,T\}}\theta_{m,s | r_{\ell,13}}}{\max_{s \in \{C,T\}}\omega_{s | r_{\ell,13}}-\min_{s \in \{C,T\}}\omega_{s | r_{\ell,13}}},\\ b &=\min_{s \in \{C,T\}}\theta_{m,s | r_{\ell,13}}-a\cdot\min_{s \in \{C,T\}}\omega_{s | r_{\ell,13}} \end{aligned} $$

The parameters for the specificity of RVDs from $\mathcal {R}_{1}$ to bind 5mC result from the following scaling:

$$\theta^{M}_{m,5mC | r_{\ell}}=\omega_{5mC | r_{\ell}}\cdot a + b - \theta^{M}_{m,5mC | r_{\ell,13}}, $$

with

$$\begin{aligned} a &= \frac{\max_{s \in \{C,T\}}\theta_{m,s | r_{\ell},r_{\ell,13}}-\min_{s \in \{C,T\}}\theta_{m,s | r_{\ell},r_{\ell,13}}}{\max_{s \in \{C,T\}}\omega_{s | r_{\ell}}-\min_{s \in \{C,T\}}\omega_{s | r_{\ell}}},\\ b &=\min_{s \in \{C,T\}}\theta_{m,s | r_{\ell},r_{\ell,13}}-a\cdot\min_{s \in \{C,T\}}\omega_{s | r_{\ell}},\\ &\qquad\theta_{m,T| r_{\ell},r_{\ell,13}}=\theta_{m,T| r_{\ell}}+\theta_{m,T|r_{\ell,13}},\\ &\qquad\theta_{m,C| r_{\ell},r_{\ell,13}}=\theta_{m,C| r_{\ell}}+\theta_{m,C|r_{\ell,13}} \end{aligned} $$

Here, we consider only values measured for 5mC but not those measured for 5hmC for two reasons. First, the plant genome contains much less 5hmC than 5mC [42, 43]. Second, it is not possible to distinguish between 5mC and 5hmC in bisulfite sequencing. We also decided not to calculate the average over both measurements, since the specificity of both differs substantially for some RVDs.

**Fig. 1:**
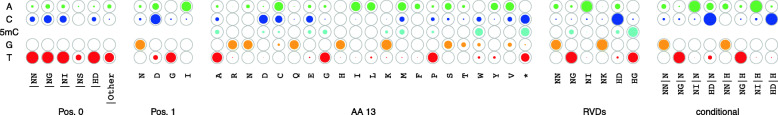
Parameters of the EpiTALE model. Parameters are represented by circles that are arranged in rows according to the bound nucleotide including 5mC. Column labels indicate the RVD, 13th AA, or combination of RVD and previous 12th AA that a parameter depends on. The outer circles are filled by a coloured circle proportional to the corresponding specificity parameters, where different colours are used for the four nucleotides and 5mC, respectively. For instance, the RVD ’HD’ has a strong preference for C, while A, 5mC and T are bound in decreasing preference, and G is a clear mismatch for ’HD’. There are no separate parameters for methylated cytosines for the sub-model at position 0, position 1 and the “conditional” sub-model, as there are no sufficient data available yet

A visualization of the model parameters of EpiTALE including the parameters for 5mC is shown in Fig. 1. Several of the thirteenth amino acids and several of the common RVDs show large differences in specificity between an unmethylated and a methylated cytosine.

### Accessibility filter

Target boxes predicted by EpiTALE may further be filtered for chromatin accessibility. For each predicted target sequence, a window from 300 bp upstream to 50 bp downstream of the target box is checked for an overlapping peak within the peaks determined by JAMM [34] from chromatin accessibility data.

As an additional filter criterion, the number *n* of positions that correspond to at least one 5’-end of a read within a defined region around the predicted target box is considered. For promoterome-wide predictions, this region corresponds to the complete promoter sequence (-300 bp to +200 bp relative to TSS [18]) that is scanned for putative TALE target boxes. In genome-wide predictions, the complete genomic sequence of the host plant is scanned for putative TALE target boxes and the scanning process is annotation-agnostic by concept. Hence, an anchor point similar to the TSS in promoterome-wide predictions is lacking for genome-wide predictions. As a proxy, we use the position of the predicted target box in this case, and consider a window from -300 bp to +200 bp around the predicted target box, instead.

If there is an overlapping peak *or* the coverage filter criterion is fulfilled, we consider the target box as accessible.

### Prediction of TALE target boxes

The basic procedure of score calculation in a sliding window along the input sequences remains as described previously [19]. Additionally, we use the scaled parameters for methylation specificity and the methylation levels from WGBS data for promoterome-wide TALE target prediction. Here, we compare promoterome-wide predictions with and without methylation information and we perform these studies for each TALE of 3 *Xoo* strains and 10 *Xoc* strains (Supplementary File F).

In addition, DNase-seq and ATAC-seq data were used to check the predicted targets for accessibility and to derive a filter criterion based on the predictions for TALEs from the 3 *Xoo* strains. We then applied this fixed filter criterion to the predictions for TALEs from the 10 *Xoc* strains.

### Genome-wide predictions & filtering

With EpiTALE, we perform genome-wide predictions in the *Oryza sativa* Nipponbare genome (MSU7) including methylation information and the filter based on chromatin accessibility. For the resulting top 100 predictions of each TALE, we use above mentioned RNA-seq data to search for a differentially expressed region near the putative target box using DerTALE, as described previously [19]. We visualize the resulting profiles with an auxiliary R script, which plots the RNA-seq profile surrounding the putative target box and uses gff3 files to display known genes overlapping with the profile. Here, we use the MSU7 annotation (http://rice.uga.edu/pub/data/Eukaryotic_Projects/o_sativa/annotation_dbs/pseudomolecules/version_7.0/all.dir/all.gff3). For differentially expressed regions with no overlapping annotated gene, it might be that a true gene locus is missing from the existing annotation, even for high-quality annotations like MSU7. Especially the annotation of non-coding RNAs may be incomplete for non-model plants. For this reason, we use blastx and blastn to search for similar sequences in the non-redundant protein sequence (nr) database and the reference RNA sequences (refseq_rna) database using NCBI BLAST+ version 2.7.1 [44] (ftp://ftp.ncbi.nlm.nih.gov/blast/executables/blast+/LATEST/). High-quality BLAST matches in other organisms may provide indication that the differentially expressed region is indeed a functional gene. Furthermore, functional annotations from these organisms may also give insights into the role of such genes in the infections process.

### Evaluation of prediction results

To compare the impact of the two epigenetic features, we evaluate the following prediction variants: original PrediTALE model without epigenetic features (P), EpiTALE with consideration of methylation (P + Methyl), EpiTALE with filtering based on the accessibility filter criterion (P + Access) and EpiTALE with methylation and accessibility filtering (P + Methyl + Access).

We compare the performance of these 4 variants for the above mentioned *Xoo* and *Xoc* strains based on the corresponding RNA-seq infection studies. For benchmarking based on differentially expressed genes, we consider a promoter region 300 bp upstream of the transcription start site to 200 bp downstream of the transcription start site or until the start codon as described previously [18, 19]. In addition, we apply the previous tools for TALE target prediction Target Finder [15, 16], Talvez [17], and TALgetter [18] using default parameters to the extracted promoter sequences providing the RVD sequences of the TALEs present in the respective *Xanthomonas* strain. In case of multiple predictions per gene, only the prediction yielding the best prediction score is considered.

The use of RNA-Seq data from inoculation studies to evaluate the predictions entails two problems: First, when plant tissue is inoculated with a *Xanthomonas* strain, multiple TALEs lead to differential gene expression. Hence, it is not possible to clearly assign differentially expressed genes to a particular TALE. The RVD sequences of the TALEs of the *Xanthomonas* strains studied are given in F. Secondly, it is not clear whether a gene was up-regulated by the binding of a TALE to its promoter or by secondary effects triggered through inoculation with the *Xanthomonas* strain.

In order to compare previous tools and the 4 EpiTALE prediction variants mentioned above, we proceed in analogy to the previous comparison of PrediTALE with alternative approaches [19]: We vary the number *t* of predictions considered per TALE between 1 and 50, i.e., we only consider the *t* predictions with the largest prediction scores for each TALE. Given the list of predicted target genes corresponding to the top *t* predictions per TALE, we may check each of these target genes for up-regulation in the infection experiment.

Specifically, we define all genes as true positives (TPs) that are up-regulated in the RNA-seq data relative to control and have a predicted target box within the promoter. We define those genes as false positives (FPs) that are not up-regulated after inoculation, but have a predicted target box in their promoter. The definition of false negatives is not clearly possible, since up-regulated genes without a predicted target box could be indirect target genes. Following this procedure, we record the number of TPs given each cutoff *t* on the number of predictions per TALE.

### Availability

The EpiTALE suite is available as a JavaFX-based stand-alone application with graphical user interface and as command line application under http://jstacs.de/index.php/EpiTALE. A minimal example for testing is available from zenodo at https://www.doi.org/10.5281/zenodo.4749294. Source code is available from https://github.com/Jstacs/Jstacs in package projects.tals.epigenetic.

The EpiTALE suite contains tools, that (i) convert BedMethyl files to Bismark format, (ii) merge two Bismark files, (iii) compute a coverage pileup of 5’ ends of mapped reads from an DNase-seq or ATAC-seq experiment, (iv) normalize the coverage pileup relative to the mean of a 10000 bp sliding window, (v) convert methylation data (Bismark files) and chromatin accessibility data (coverage pileup and/or narrowPeak file) to promoter coordinates and (vi) predict TALE target boxes with optional epigenetic input within genomic or promoter sequences.

## Results and discussion

It has been shown previously [9, 10] that the specificity of RVDs for methylated cytosines differs from unmethylated cytosines. In EpiTALE, we include separate specificities for methylated cytosines (cf. Methods) and allow for considering methylation levels along the input sequences in TALE target prediction. In addition, binding of TALEs to their target box may be influenced by local DNA accessibility [12, 14]. Hence, EpiTALE implements an accessibility-based filter on predictions of target boxes.

The potential improvements achieved by these extensions may be twofold. First, introducing separate specificity parameters for methylated cytosines may yield more meaningful prediction scores, ideally improving the ranks of true positive predictions of target boxes. Second, an accessibility-based filter that predominantly excludes previous false positive predictions of target boxes may achieve a better enrichment of true positives among the top predictions of EpiTALE. In a practical scenario where only a limited number of predictions may be validated in wet-lab experiments, improved ranks of true positive predictions and exclusion of false positive predictions from the top ranks have the potential to increase the rate of validated and potential virulence targets.

In the following sections, we first assess the stability of prediction performance regarding different filtering criteria on the accessibility data. We then present a comprehensive benchmarking experiment including different variants of EpiTALE considering epigenetic features, where we also include previously published tools for TALE target prediction as a reference. We further investigate to which extent epigenetic features help to improve the ranks of true positive predictions, and finally apply EpiTALE including methylation information and the accessibility-based filter to yield genome-wide predictions of TALE targets.

### Performance evaluation of different accessibility filter parameters on DNase dataset

In this section, we benchmark the effect of different filters based on chromatin accessibility applied to EpiTALE predictions – at this stage still without using methylation information. To this end, we test different filtering thresholds for predicting target boxes of the TALEs present in 3 *Xoo* strains, and we evaluate the chosen filter criteria on independent data for 10 *Xoc* strains. Here, we need to resort to a rather indirect assessment of predictions based on RNA-seq data, because currently ChIP-seq data for TALEs are lacking.

Hence, predictions are evaluated based on RNA-seq data from infection experiments of these *Xoo* and *Xoc* strains compared to mock control. Specifically, we consider a predicted target gene as true positive (TP) if its promoter contains a predicted TALE target box and this gene is up-regulated in the infection experiment. We consider a predicted target gene as false positive (FP) if its promoter contains a predicted TALE target box but is not up-regulated in the infection experiment (cf. Methods: Evaluation of prediction results).

Supplementary Figure S2 shows violin plots of the accessibility values for the three accessibility datasets considered. Here, we compare the accessibility of TP predictions compared with FP predictions according to RNA-seq data. Predictions are generated for TALEs present in *Xoo* and the *Xoc* strains and chromatin accessibility is summarized per predicted target box as the fraction of promoter positions that are covered by at least one 5’ end of a read. We always consider the window starting 300 bp upstream and ending 50 bp downstream the target box in the strand orientation of the downstream gene.

The violin plot of the DNase dataset shows a visible although small difference in accessibility between TP and FP targets for both *Xoo* and *Xoc*. The two ATAC-seq datasets, however, show substantially smaller differences with almost identical median values and generally low fraction of covered promoter positions. The p-values of a Wilcoxon rank sum test show a statistically significant difference between TP and FP predictions for all three datasets and strains, except for the ATAC1 dataset in case of the *Xoc* strains. These ATAC-seq datasets are likely of limited use for filtering TALE target predictions. Hence, we focus on the DNase-seq data in the following analyses, and provide results using the ATAC2 dataset as supplementary figures. The reason that the two ATAC-seq datasets are less suited for filtering TALE target predictions may be the relatively low genomic coverage with ATAC-seq reads but also different experimental conditions when collecting these publicly available ATAC-seq data. Plants at different life stages and grown under different greenhouse conditions may have different accessibility profiles. For the DNase-seq data, this issue seems to be less severe, although DNase data have been collected for rice seedlings. We speculate that high-coverage ATAC-seq data collected under the same conditions as for the infection experiments might still be informative for TALE target prediction.

The accessibility filter criterion consists of two parts: A putative target box from the initial predictions survives the filter if it has an overlapping peak of chromatin accessibility within the window from 300 bp upstream to 50 bp downstream of the target box. A putative target box also survives the filter if at least *n* positions within the complete promoter correspond to at least one 5’ end of a read.

The performance of EpiTALE with a filter based on the DNase-seq dataset compared with the original PrediTALE neglecting chromatin accessibility is shown in Supplementary Figure S3 for three *Xoo* strains. Here, different thresholds *n* on the number of positions covered by the 5’ end of at least one read are considered, and the number of true positive (TP) target boxes is plotted against the number of predictions allowed per TALE, with a rank cutoff from 1 to 50. To ensure comparability to the original PrediTALE publication [19], we use the same type of performance plots with the same rank cutoffs and the same definition for differentially up-regulated genes caused by the respective strains in the RNA-seq infection studies. Briefly, we consider those genes as putatively up-regulated by TALEs that have an uncorrected p-value below 0.05 in the RNA-seq infection studies of the 3 *Xoo* strains and are at least 2-fold up-regulated. Here, we consider these rather relaxed criteria, as we want to avoid predictions to be erroneously considered false positives due to the larger variation at the early time point after infection (24 h) for the *Xoo* strains.

Using the accessibility filter, a threshold of *n*=30 yields the largest area under the curve of TP predictions (AUC-TP) for the strains ICMP 3125^T^ and PXO83. In case of ICMP 3125^T^, filtering with this threshold for any rank cutoff shows improved or at least identical performance as PrediTALE without filtering, and a larger improvement than any other filter threshold tested. For PXO83, the same effect can be observed, where only when considering the top 3 predictions per TALE, a threshold of *n*=35 results in one additional TP. For PXO142, accessibility filtering with a threshold of *n*=30 within a range of 1 to 30 total predictions per TALE increases or at least retains the number of TP predictions. For higher rank cutoffs, the filtering results in one TP less than for the unfiltered version. Thus, a threshold of *n*=30 leads to a reduction of TPs only in rare cases and mostly leads to an increase in the number of TPs within the top predictions. In complete analogy, Supplementary Figure S7 shows performance plots for the 10 *Xoc* strains. As described previously [19], we consider standard criteria for differentially expressed genes (*q*−*value*<0.01, log2 fold change > 2) for the *Xoc* strains, as these RNA-seq data have been collected 48 h after infection. For 8 of the 10 strains, the accessibility filtering based on the DNase dataset results in a higher AUC-TP for each of the 6 thresholds. However, for 7 of these 8 strains a threshold of *n*=25 results in the highest AUC-TP. The threshold of *n*=30 chosen from the *Xoo* datasets does not result in the optimal result for *Xoc*, but performs substantially better than the original PrediTALE version without filtering. Filtering based on the DNase-seq dataset works slightly worse only for strains CFBP7331 and CFBP7341.

For the predictions of the top 50 target boxes of each TALE of the *Xoo* and *Xoc* strains considered, the proportion of TP and FP target boxes that pass the accessibility filter is shown in Supplementary Figure S9. The TP target boxes are usually accessible according to the accessibility filter criterion with a threshold of *n*=30. FP target boxes in turn are rather filtered out as they are occasionally inaccessible.

### Performance of EpiTALE model considering epigenetic DNA modifications

In this section, we further investigate the effect of including methylation-specific parameters into the EpiTALE model, and its combination with the accessibility filter studied in the previous section. Specifically, we consider four modelling alternatives: i) the original PrediTALE model (P), ii) the EpiTALE model including specificities for methylated cytosines (P + Methyl), iii) the PrediTALE model combined with the accessibility filter (P + Access), and iv) the EpiTALE model combined with the accessibility filter (P + Methyl + Access). As a reference, we further include tools for TALE target prediction that have been published previously, namely Target Finder [15, 16], Talvez [17], and TALgetter [18]. Neither of these three tools considers epigenetic features when predicting TALE targets.

**Fig. 2:**
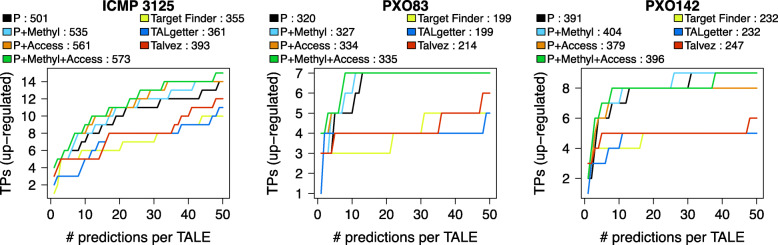
EpiTALE performance evaluation for three *Xoo* strains considering epigenetic features. We plot the number of predicted target genes that are also up-regulated in the infection (true positives, TPs) against the number of predicted target sites per TALE for PrediTALE (P) and three EpiTALE variants including only methylation information (P+Methyl), only filtering based on chromatin accessibility (P+Access), or a combination of both (P+Methyl+Access). As a reference, we include the previous TALE target prediction tools Target Finder, Talvez, and TALgetter that do not consider epigentic features. In the legends, we further report the area under the curve of TP predictions (AUC-TP) for the three previous tools, PrediTALE and the individual EpiTALE variants

The results of the performance evaluation of previous tools and these four EpiTALE variants for TALE target prediction of *Xoo* TALEs are shown in Fig. 2. Here, accessibility is determined based on the DNase dataset. The number of true positive predictions is improved by either of the epigenetic features for the strains ICMP 3125^T^ and PXO83, where the improvement due to the accessibility filter is more pronounced than the improvement due to including methylation levels into the EpiTALE model. For both strains, performance is further increased by combining both epigenetic features. For PXO142, the accessibility filter alone leads to slightly decreased prediction performance, whereas methylation information alone as well as the combination of both epigenetic features leads to a slight improvement compared with the original PrediTALE variant. All variants of EpiTALE yield a considerable improved compared with previous tools for TALE target prediction.

**Fig. 3:**
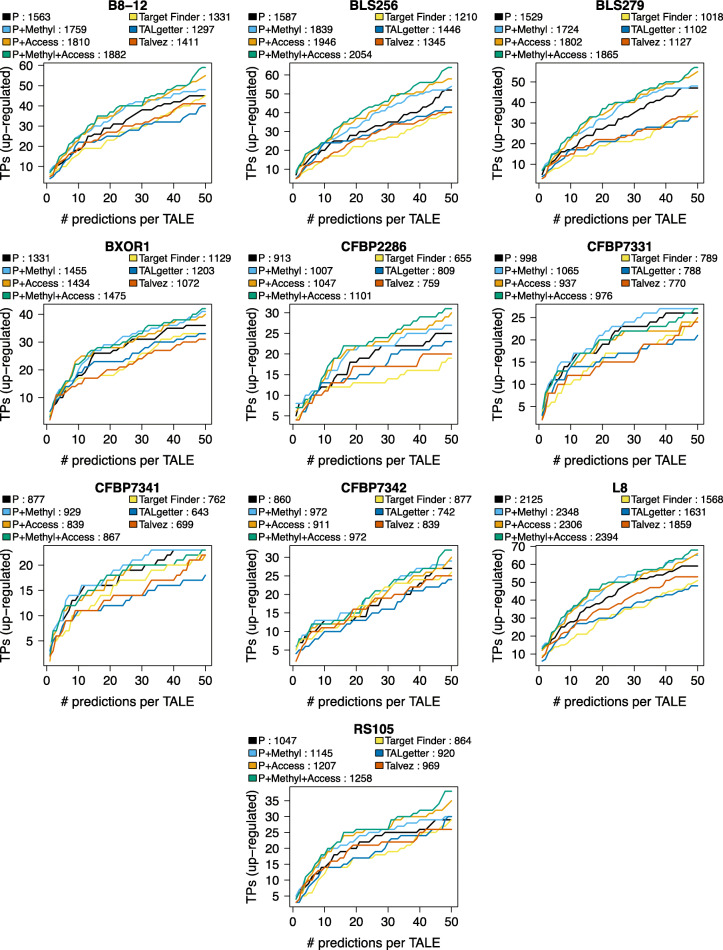
EpiTALE performance evaluation for ten *Xoc* strains considering epigenetic features. We plot the number of predicted target genes that are also up-regulated in the infection (true positives, TPs) against the number of predicted target sites per TALE for PrediTALE (P) and three EpiTALE variants including only methylation information (P+Methyl), only filtering based on chromatin accessibility (P+Access), or a combination of both (P+Methyl+Access). As a reference, we include the previous TALE target prediction tools Target Finder, Talvez, and TALgetter that do not consider epigenetic features. In the legends, we further report the area under the curve of TP predictions (AUC-TP) for the three previous tools, PrediTALE and the individual EpiTALE variants

The results for the *Xoc* strains are shown in Fig. 3. For 8 of the 10 strains, methylation information, filtering according to target box accessibility, and the combination of both epigenetic features lead to a clear increase of AUC-TP compared to PrediTALE without these features. For CFBP7331 and CFBP7341, only considering the methylation information leads to an improvement, because the accessibility criterion for these two strains is too strict in some cases and a few TP target boxes are determined to be inaccessible. However, the EpiTALE variant considering methylation and accessibility still yields more accurate predictions than previous tools for TALE target prediction excepting PrediTALE.

The performance based on the accessibility dataset ATAC2 for 3 *Xoo* strains is presented in Supplementary Figure S5. In this case, the performance of the EpiTALE variants for which accessibility is used for filtering is substantially lower. In order to rule out the possibility that the decrease in performance is simply due to the chosen filtering criteria, we tested different thresholds for this dataset as presented in Supplementary Figure S6. However, none of the thresholds considered leads to restoring the performance of the original PrediTALE variant.

### Considering epigenetic features improves ranks of true positive targets

In this section, we focus on the top 20 predictions of the four EpiTALE variants for three *Xoo* and ten *Xoc* strains that also show upregulation after infection with the respective strains. The complete list of true positive predictions is given in Supplementary Table C, and the subset of predictions for *Xoo* strains is provided in Table 1. For each of the 3 *Xoo* strains, all three EpiTALE variants including epigenetic features mostly yield an improvement of the rank of the true positive target gene compared with the original PrediTALE variant without epigenetic features. The strongest rank improvement is almost always achieved by the EpiTALE variant that considers methylation of the target box as well as its accessibility. However, an improvement can often be observed already when considering only one of the epigenetic features.

**Table 1:** Putative TALE target genes that are among the top 20 predictions per TALE for any of the four approaches

Gene	lfc	P	P+Methyl	P+Access	P+Methyl+Access	Annotation
**ICMP 3125**^**T**^						
Os02g06670	3.815	TalBA8 (1)	TalBA8 (1)	TalBA8 (1)	TalBA8 (1)	retrotransposon protein
Os09g29820 [6, 45]	2.819	TalAR13 (2)	TalAR13 (2)	TalAR13 (2)	TalAR13 (2)	OsTFX1 - bZIP transcription factor
Os03g51760	2.734	TalAD22 (9)	TalAD22 (7)	TalAD22 (6)	TalAD22 (6)	OsFBX109 - F-box protein
Os04g05050	2.221	TalAB16 (11)	TalAB16 (8)	TalAB16 (7);	TalAB16 (7)	pectate lyase
Os01g40290 [6]	1.894	TalAA15 (1)	TalAA15 (1)	TalAA15 (1)	TalAA15 (1)	expressed protein
Os05g45070	1.704	TalAO15 (15)	TalAO15 (13)	TalAO15 (11)	TalAO15 (10)	harpin-induced protein 1
Os11g26790 [17]	1.695	TalAH11 (1)	TalAH11 (1)	TalAH11 (1)	TalAH11 (1)	dehydrin
Os06g03710	1.591	TalES1 (19)	TalES1 (17)	TalES1 (13)	TalES1 (12)	DELLA protein SLR1
Os01g73890 [6, 46]	1.079	TalBM2 (1)	TalBM2 (1)	TalBM2 (1)	TalBM2 (1)	transcription initiation factor IIA gamma
Os10g28240	0.918	TalAR13 (6)	TalAR13 (6)	TalAR13 (4)	TalAR13 (4)	calcium-transporting ATPase
Os09g07460	0.746	TalBA8 (22)	TalBA8 (19)	TalBA8 (18)	TalBA8 (17)	kelch repeat protein
**PXO142**						
Os02g49350	5.163	TalBH2 (8)	TalBH2 (5)	TalBH2 (7)	TalBH2 (5)	plastocyanin-like
Os03g09150	2.530	TalBH2 (4)	TalBH2 (3)	TalBH2 (4)	TalBH2 (3)	pumilio-family RNA binding
Os11g31190	2.514	TalBH2 (3)	TalBH2 (2)	TalBH2 (3)	TalBH2 (2)	SWEET14 (nodulin MtN3)
Os09g29820 [6, 45]	2.272	TalAR14 (3)	TalAR14 (2)	TalAR14 (2)	TalAR14 (2)	OsTFX1 - bZIP transcription factor
Os03g51760	1.368	TalAD23 (13)	TalAD23 (11)	TalAD23 (8)	TalAD23 (8)	OsFBX109 - F-box protein
Os01g40290 [6]	0.887	TalAA16 (1)	TalAA16 (1)	TalAA16 (1)	TalAA16 (1)	expressed protein
Os06g29790 [18]	0.833	TalAO16 (4)	TalAO16 (4)	TalAO16 (3)	TalAO16 (3)	phosphate transporter 1
Os07g06970 [6]	0.824	TalAP15 (1)	TalAP15 (1)	TalAP15 (1)	TalAP15 (1)	HEN1
**PXO83**						
Os09g29820 [6, 45]	2.82	TalAR3 (5)	TalAR3 (4)	TalAR3 (1)	TalAR3 (1)	OsTFX1 - bZIP transcription factor
Os02g06670	2.74	TalAR3 (83); TalBA2 (1)	TalAR3 (74); TalBA2 (1)	TalAR3 (52); TalBA2 (1)	TalAR3 (48); TalBA2 (1)	retrotransposon protein
Os03g51760	1.91	TalAD5 (13)	TalAD5 (11)	TalAD5 (8)	TalAD5 (8)	OsFBX109 - F-box protein
Os04g19960	1.70	TalAC5 (1)	TalAC5 (1)	TalAC5 (1)	TalAC5 (1)	retrotransposon protein
Os04g05050	1.62	TalAB5 (11)	TalAB5 (8)	TalAB5 (7)	TalAB5 (7)	pectate lyase
Os07g06970 [6]	1.40	TalAP3 (1)	TalAP3 (1)	TalAP3 (1)	TalAP3 (1)	HEN1
Os03g03034	1.18	TalAQ3 (5)	TalAQ3 (4)	TalAQ3 (4)	TalAB5 (97); TalAQ3 (3)	flavonol synthase

For each *Xoo* strain, we list the gene ID (MSU7) and the log fold change (lfc) in the corresponding RNA-seq experiment. For each of the four EpiTALE variants, we further list the TALE(s), for which a gene has been predicted as a target and in parentheses the corresponding prediction rank. For known TALE target genes, we provide the corresponding reference after the gene ID

The gene Os09g07460, coding for a kelch repeat protein, is among the top 20 predictions for TalBA8 for all three EpiTALE variants considering epigenetic features. This gene has not been among the top 20 predictions of the original PrediTALE variant, but has been reported by Talvez [17, 19].

Regarding target boxes predicted for TALEs from ten *Xoc* strains (cf. Supplementary Table C), all three EpiTALE variants including epigenetic features in the majority of cases either yield an improved or an unchanged prediction rank for true positive genes.

The accessibility filter criterion appears to be inappropriate for some putative target boxes upstream of true positive target genes, which are determined to be inaccessible, although they are upregulated in the RNA-seq experiments. This applies to the putative target box in the promoter of Os01g52130 for TalBF members from *Xoc* strains B8-12, BLS256, BLS279, CFBP2286, BXOR1, CFBP7331, CFPB7341, CFPB7342, L8, RS105; the putative target box upstream of Os02g06130 for TalAF from B8-12 and L8; the putative target box upstream of Os07g01490 for TalBD from B8-12, BLS256, BLS279, BXOR1, L8; the putative target box upstream of Os07g03279 for TalBE from BXOR1, CFBP7331, CFPB7341; the putative target box upstream of Os03g22020 for TalBU from CFBP7331 and the putative target box upstream of Os12g06930 for TalBI from CFPB7342.

Out of 323 true positive target boxes, the majority of 212 target boxes, however, obtains an improved rank when considering both epigenetic features, while the rank of 87 target boxes remains unchanges compared with the original PrediTALE variant. Among the target genes with an improved prediction rank are well known TALE targets like Os07g06970 coding for HEN1, but also promising novel candidates like Os03g53800 a beta-glucosidase precursor.

Both the methylation information and chromatin accessibility considered in this study have been derived from publicly available datasets that have been collected for different purposes and scientific questions. Hence, these have been determined under different conditions, e.g., from different plants at a different life stage than for the infection studies that are represented by the RNA-seq data. On the one hand, this may explain both the lowered ranks of the above-mentioned true positive target genes when considering methylation information, but also target genes that are up-regulated in the infection studies not passing the accessibility filter. On the other hand, the widely improved prediction ranks for many of the remaining true positive target genes provide a strong indication that both types of data provide valuable information for TALE target prediction. Our results suggest that with matched WGBS and DNase-seq/ATAC-seq data of sufficient quality, the quality of computational TALE target predictions could be boosted even further.

### Genome-wide TALE target prediction considering DNA methylation and chromatin accessibility

Independently of existing gene annotations, we performed genome-wide predictions of TALE target boxes in *Oryza sativa* Nipponbare (MSU7) for 3 *Xoo* and 10 *Xoc* strains using the EpiTALE version with methylation and DNase accessibility data. As accessibility filter, we select regions around the binding site based on criteria that are similar to the promoter setting. Either a peak of chromatin accessibility must be present in a region from -300 bp to +50 bp relative to the target box or at least 30 positions within the window from -300 bp to +200 bp relative to the target box must be covered by the 5’-end of at least one DNase-seq read. The latter criterion implies that at least 30 DNase-seq reads must overlap this window around the target box, but might even require a larger number of DNase-seq reads if the 5’-ends of multiple reads map to the same location.

To determine differentially expressed regions near predicted target boxes, we use our tool DerTALE [19] and the mapped RNA-Seq from above-mentioned infection studies. For DerTALE we use the same settings as in the original PrediTALE publication [19]. Briefly, we search for differentially expressed regions of at least 300 bp within a region of ± 3000 bp around the top 100 predicted target boxes of each TALE.

**Fig. 4:**
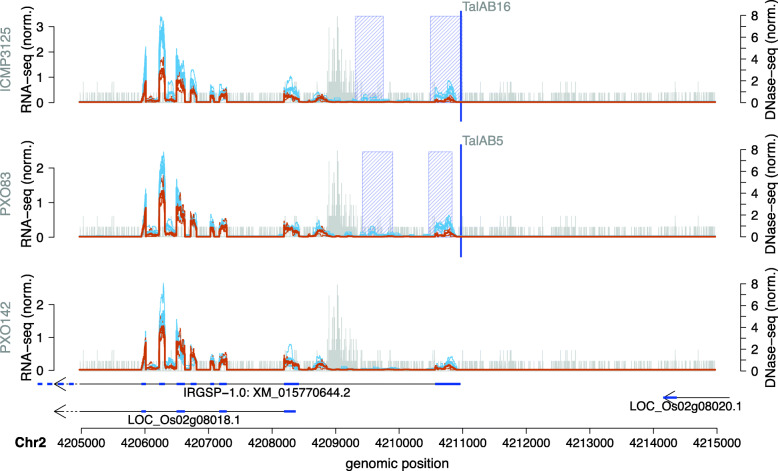
Genome-wide predictions of TalAB in *Oryza sativa* Nipponbare profile for 3 *Xoo* strains in the area of the TalAB target box. RNA-seq coverage of 3 replicates after inoculation (thin blue lines) are compared with the RNA-seq coverage of 3 replicates of mock control (thin brown lines). In addition, we plot the average coverage of individual replicates after inoculation as a thick blue line and the average coverage of individual replicates of mock control as a thick brown line. The blue shaded boxes mark the differentially expressed regions. The arrows under the profiles reflect the MSU7 annotation within the genomic region. The genomic position of the TALE target box is marked by a vertical blue line. Vertical grey bars indicate the number and 5’-position of reads in the DNase data

Genome-wide prediction shows that for 16 *Xoo* TALEs, differential expressed regions are close to at least one predicted target box. In total, we obtain 20 of such target boxes (complete list in Supplementary Table D), of which 13 have also been observed in the previous prediction limited to promoters. Among these 20 target boxes, 18 have already been reported in the original PrediTALE publication [19]. By using the two epigenetic features in the EpiTALE variant, we obtain 2 novel target boxes near differentially expressed regions. Figure 4 presents the RNA-seq profile in the region of a target box predicted for members of family TalAB on chromosome 2. The putative target boxes of TalAB5 (PXO83) and TalAB16 (ICMP 3125^T^) are identical and do not overlap with a gene annotation known from MSU7. We extracted the sequences under the differentially expressed regions, and first compared them against the NCBI protein database ‘nr’ using blastx but received no matching result. We additionally compared these sequences against the NCBI reference RNA sequences (‘refseq_rna’) using blastn, which resulted in a predicted mRNA, coding for a calcium-transporting ATPase (XM_015770644.2).

However, one putative target box reported from genome-wide predictions in the original PrediTALE publication (Os04g05050) appears on a lower rank due to methylation of the target box, which might be caused by non-matching experimental conditions as discussed previously.

**Fig. 5:**
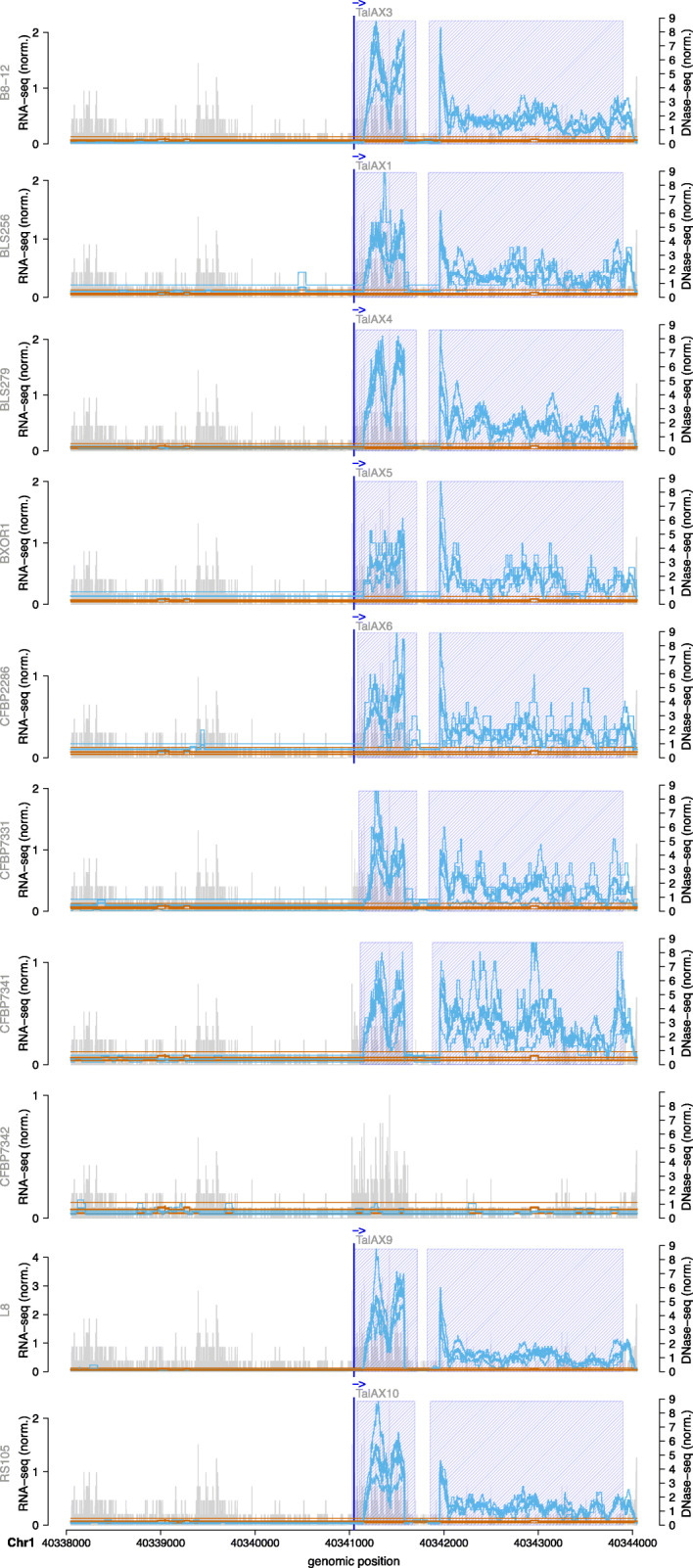
Genome-wide predictions of TalAX in *Oryza sativa* Nipponbare profile for 10 *Xoc* strains in the area of the TalAX target box. RNA-seq coverage of 3 replicates after inoculation (thin blue lines) are compared with the RNA-seq coverage of 3 replicates of mock control (thin brown lines). In addition, we plot the average coverage of individual replicates after inoculation as a thick blue line and the average coverage of individual replicates of mock control as a thick brown line. The blue shaded boxes mark the differentially expressed regions. The genomic position of the TALE target box is marked by a vertical blue line. Vertical grey bars indicate the number and 5’-position of reads in the DNase data

A complete list of the genome-wide predictions of TALEs from the ten *Xoc* strains is given in Supplementary Table E, of which we select two examples for a detailed discussion. The first of these is a predicted target box of members of the TalAX family located on chromosome 1. Members of TalAX are present in all ten *Xoc* strains. The corresponding class tree is shown in Supplementary Figure S11 and the RNA-seq profile around the putative target box is provided in Fig. 5. Close to the putative target box is a differentially expressed region that has no overlapping MSU7 gene annotation. For 7 of the 10 strains, the predicted target box is among the top 100 predictions. For strains CFBP7331 and CFPB7341, this target box appears only in the top 200 prediction due to differences in the RVD sequence of the TalAX members present in these strains. However, the RNA-seq data suggest that these TALEs are still capable of activating downstream expression since a differential region is detected for these strains as well. TalAX2 from CFBP7342 deviates even further from the RVD composition of the remaining strains, and no target box in this region was predicted for TalAX2. In agreement with this prediction, we do not observe a differentially expressed region after *Xoc* CFBP7342 infection. For the sequences under this differentially expressed region, database search using blastx and blastn against ’nr’ and ’refseq_rna’, respectively, did not result in a match.

**Fig. 6:**
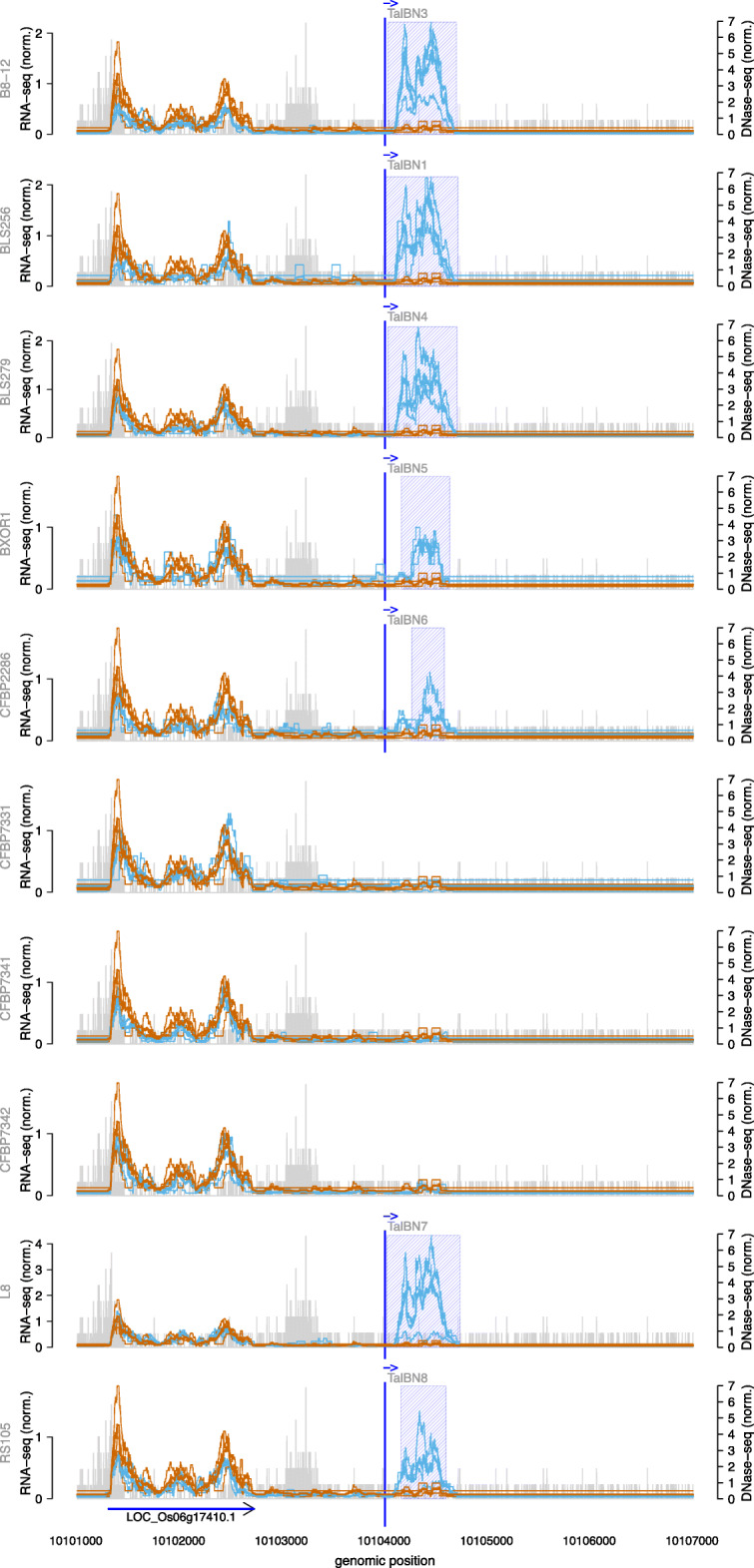
Genome-wide predictions of TalBN in *Oryza sativa* Nipponbare profile for 10 *Xoc* strains in the area of the TalBN target box. RNA-seq coverage of 3 replicates after inoculation (thin blue lines) are compared with the RNA-seq coverage of 3 replicates of mock control (thin brown lines). In addition, we plot the average coverage of individual replicates after inoculation as a thick blue line and the average coverage of individual replicates of mock control as a thick brown line. The blue shaded boxes mark the differentially expressed regions. The arrows under the profiles reflect the MSU7 annotation within the genomic region. The genomic position of the TALE target box is marked by a vertical blue line. Vertical grey bars indicate the number and 5’-position of reads in the DNase data

As a second example, we discuss a putative target box on chromosome 6 for members of the TalBN class present in 8 of 10 *Xoc* strains. The corresponding class tree is shown in Supplementary Figure S12 and the RNA-seq profile around the binding site is presented in Fig. 6. The TalBN members from 7 of the 8 strains have identical RVD sequences, whereas TalBN2 of CFBP7342 show one difference in RVD sequence. This target box on chromosome 6 is among the top 100 predictions only for these 7 TalBN members and DerTALE report a differentially expressed region after infection with these strains. The remaining TalBN members (TalBN2 of CFBP7342) has no putative target box among the top 100 predictions at this position, and the region shows no differential expression as well as for the 2 strains with no TalBN member (CFBP7341, CFBP7331). This indicates that this differentially expressed region may be caused by TalBN members of the strains with the putative target site. The differentially expressed region does not overlap with an annotated MSU7 gene and the corresponding sequence had no matches in BLAST searches.

## Conclusions

With the goal of improving the prediction of TALE targets, we present EpiTALE, an extended version of PrediTALE including epigenetic features. Both, methylation levels and the chromatin accessibility around putative target sites have a decisive impact on the likelihood of being bound by a TALE. Even if a putative target box matches the specificity of the RVDs of a TALE, inaccessibility of the respective chromatin may inhibit binding and thus inhibit activation of the transcription of the downstream gene [47, 48]. We demonstrate that for the prediction of TALE target boxes, the consideration of the epigenetic state of rice plants leads to an improved quality of TALE target predictions by EpiTALE compared with PrediTALE and three other previous tools for TALE target prediction. For many true positive *Xoo* and *Xoc* target boxes, EpiTALE yielded improved prediction ranks of true positive targets compared with the original PrediTALE variant. Nevertheless, there are still false positive predictions and we suggest an experimental verification of novel targets.

We perform promoter-wide and genome-wide predictions and find several predictions common to both approaches, but we also find target boxes upstream of differentially expressed regions in RNA-seq infection studies that do not overlap with a currently annotated gene.

The use of the epigenetic features is optional for the user. Depending on the availability of data, only methylation and/or chromatin accessibility data can be provided to EpiTALE to improve target prediction. In our study, the strongest improvement in accuracy was achieved by considering both epigenetic features in EpiTALE. Our results suggest that collecting condition-matched WGBS and DNase-seq/ATAC-seq data may further improve the quality of computational TALE target predictions. The EpiTALE suite presented here provides the means necessary to integrate such data into TALE target prediction and is available from http://jstacs.de/index.php/EpiTALE.

## Supplementary Information

**Additional file 1** PDF file integrating Supplementary Figures S1 – S12.

**Additional file 2** Mapping statistics of ATAC and DNase-seq data.

**Additional file 3** List of positive and negative targets for *Xoo* and *Xoc*.

**Additional file 4** Complete list of top 20 predictions for all four varaiants and *Xoo* and *Xoc* strains.

**Additional file 5** Genome-wide prediction for three *Xoo* strains.

**Additional file 6** Genome-wide prediction for ten *Xoc* strains.

**Additional file 7** RVD sequences of all *Xoo* and *Xoc* TALEs considered in this manuscript.

## Data Availability

Public whole genome bisulfite sequence data have been obtained from the European Nucleotide Archive (https://www.ebi.ac.uk/ena) and are available under run accessions SRR3485276 and SRR3485277. The genomic sequence of rice (*Oryza sativa* ssp. *japonica* Nipponbare) has been obtained from http://rice.uga.edu/pub/data/Eukaryotic_Projects/o_sativa/annotation_dbs/pseudomolecules/version_7.0/all.dir/. Public DNase-seq data has been obtained from NCBI Sequence Read Archive (https://www.ncbi.nlm.nih.gov/sra) and are available under accession SRX038423. Public ATAC-seq data have been obtained from the European Nucleotide Archive and are available under accessions PRJNA305365 and PRJNA391551. Public RNA-seq data of rice plants after infection with 10 *Xanthomonas oryzae* pv. *oryzae* strains and Mock control are available from NCBI Gene Expression Omnibus (https://www.ncbi.nlm.nih.gov/geo/) under accession GSE67588. In-house RNA-seq data of rice plants after infection with 3 *Xanthomonas oryzae* pv. *oryzicola* strains and Mock control are available from the European Nucleotide Archive under accession PRJEB28127. The EpiTALE program is available as a JavaFX-based stand-alone application with graphical user interface and as command line application under http://jstacs.de/index.php/EpiTALE. Source code of the EpiTALE software is available from github at https://github.com/Jstacs/Jstacs/tree/master/projects/tals/epigenetic. A minimal example for testing EpiTALE is available from zenodo at https://www.doi.org/10.5281/zenodo.4749294.
